# Clinically Accessible Liver Fibrosis Association with CT Scan Coronary Artery Disease Beyond Other Validated Risk Predictors: The ICAP Experience

**DOI:** 10.3390/jcm14041218

**Published:** 2025-02-13

**Authors:** Belén García Izquierdo, Diego Martínez-Urbistondo, Sonsoles Guadalix, Marta Pastrana, Ana Bajo Buenestado, Inmaculada Colina, Manuel García de Yébenes, Gorka Bastarrika, José A. Páramo, Juan Carlos Pastrana

**Affiliations:** 1 Department of Endocrinology, Clínica Universidad de Navarra, 28027 Madrid, Spain; 2 Vascular Medicine Area, Clínica Universidad de Navarra, 28027 Madrid, Spain; 3 Department of Internal Medicine, Clínica Universidad de Navarra, 28027 Madrid, Spain; 4 Department of Internal Medicine, Clínica Universidad de Navarra, 31008 Pamplona, Spain; 5 Department of Cardiology, Clínica Universidad de Navarra, 28027 Madrid, Spain; 6 Department of Radiology, Clínica Universidad de Navarra, 31008 Pamplona, Spain; 7 Department of Hematology, Clínica Universidad de Navarra, 31008 Pamplona, Spain

**Keywords:** cardiovascular risk assessment, atherosclerotic disease burden, liver fibrosis, obesity, statin use

## Abstract

**Background/objectives:** Cardiovascular risk (CVR) stratification in clinical settings remains limited. This study aims to evaluate clinical parameters that could improve the identification of higher-than-expected coronary artery disease (CAD) in CT scan coronarography. **Methods:** In a cross-sectional study of asymptomatic patients from the Integrated Cardiovascular Assessment Program (ICAP), volunteers aged 40–80 without diagnosed cardiovascular disease were assessed. CVR factors like obesity, lipid and glucose profiles, liver fibrosis risk (FIB-4 ≥ 1.3), C-reactive protein, and family history of CVD were evaluated. Patients were stratified by CVR following ESC guidelines. “CVR excess” was defined as CAD-RADS ≥ 2 in low-to-moderate-risk (LMR), CAD-RADS ≥ 3 in high-risk (HR), and CAD-RADS ≥ 4 in very-high-risk (VHR) groups. **Results:** Among 219 patients (mean age 57.9 ± 1.15 years, 14% female), 43.4% were classified as LMR, 49.3% as HR, and 7.3% as VHR. “CVR excess” was observed in 18% of LMR, 15% of HR, and 19% of VHR patients. LMR patients with prior statin use and HR patients with obesity were more likely to have “CVR excess” (*p* < 0.01 and *p* < 0.05, respectively). FIB-4 modified the effect of statin use and obesity on “CVR excess” prediction (*p* for interactions < 0.05). Models including age, sex, and both interactions showed a strong discrimination for “CVR excess” in LMR and HR groups (AUROC 0.84 (95% CI 0.73–0.95) and 0.82 (95% CI 0.70–0.93), respectively). **Conclusions:** Suspected liver fibrosis combined with statin use in LMR patients and obesity in HR patients is associated with CVR excess, providing potential indications for image CAD assessment in asymptomatic patients.

## 1. Introduction

Cardiovascular disease (CVD) burden is a frequent cause of morbidity and mortality in developed countries [1]. Thus, health organizations have promoted different strategies to detect and treat patients at risk [2]. Among them, CVD risk stratification equations, which included traditional risk factors, have been an important tool to estimate individual risk based on regional adapted equations, such as SCORE2 or SCORE2-Old People (SCORE2-OP) indexes in Europe [3,4]. These scales provide a 10-year risk measure of major adverse cardiovascular event incidence. However, recent studies have shown variable results regarding the validation of these tools [5], especially in patients over 70 years old [6]. The cardiovascular (CV) risk factors considered by these SCOREs include age, sex, smoking status, non-high-density lipoprotein cholesterol (HDL-C), and systolic blood pressure. Additionally, comorbidities such as kidney disease or diabetes are associated with an increased CV risk and are included in the guidelines to improve the accuracy of patient classification [5,7].

Nevertheless, the detection and management of traditional risk factors remains insufficient for identifying patients at risk. In fact, 42.8% of women and 47.4% of men suffer from CVD due to the so-called “residual risk” according to a recently published study [8], with an improvement in discriminatory capacity when applying the Framingham scale, achieving an area under the curve (AUC) of 0.68 [9]. These findings point out unknown factors contributing to CVD risk. In this context, the identification of potential CV risk modifiers is of great interest in clinical CVD research. Multiple factors have been analyzed as potential independent markers of disease. However, the role of these additional factors remains controversial in terms of individual CVD risk prediction in clinical settings [5].

Obesity is closely linked to CVD, sharing pathophysiological mechanisms with other traditional risk factors such as dyslipidemia and insulin resistance [9]. However, clinically accessible proxies to detect patients with adipose tissue excess such as the body mass index (BMI) have not been shown to provide precision to the CV risk calculated by standardized equations [10]. Another factor which might be linked to CV risk is steatotic liver disease (SLD). Evidence suggests that SLD is a significant predictor of CV events [11,12,13,14]. A previous epidemiological study reported a CVD prevalence exceeding 35% in patients with SLD [15]. Furthermore, a large meta-analysis of 36 longitudinal studies demonstrated that SLD is independently associated with an increased long-term risk of CV events, with a further increase observed in advanced fibrosis stages (hazard ratios ranging from 1.45 to 2.50, depending on disease severity) [16]. In this regard, the new guidelines prioritize the assessment of liver fibrosis using widely available non-invasive tests in at-risk patients, with the FIB-4 (fibrosis-4) index being proposed as the standard screening tool in this population [17,18]. However, these indices primarily detect patients at risk of liver disease and lack the ability to identify individuals at CV risk in clinical settings [19]. Other factors, such as inflammation measured by C-reactive protein (CRP) or lipoprotein (a) have demonstrated the ability to detect at-risk patients at the population level but have yet to be fully integrated into clinical risk stratification [20,21,22]. Thus, evaluating the relationship between these unintegrated risk factors and their ability to detect patients with greater-than-expected subclinical atherosclerotic disease may be of interest in clinical practice.

In this context, non-invasive atherosclerotic plaque burden assessment has been revealed to enhance CV risk detection and enable personalized CV risk assessment [23]. Coronary CT angiography (CCTA) has shown to be highly accurate in diagnosing and categorizing coronary artery disease (CAD) when compared to invasive angiography [24]. The CAD-RADS (Coronary Artery Disease Reporting and Data System) classification was introduced to standardize the reporting system for patients undergoing CCTA, providing independent prognostic information through non-invasive evaluation of suspected CAD [24,25,26]. However, the population of asymptomatic patients that should undergo this procedure for an efficient CV risk reclassification is not clear yet [5].

The objective of this research is to evaluate the capacity of clinically accessible proxies to detect patients with “CV risk excess”, determined by the finding of higher-than-expected CAD using CCTA in a cohort of patients without previously diagnosed CVD, providing potential indications for image CAD assessment in asymptomatic patients.

## 2. Materials and Methods

### 2.1. Selection Criteria

A cross-sectional study was conducted with data from the Integrated Cardiovascular Assessment Program (ICAP). The ICAP is a cohort of asymptomatic patients built with the enrollment of volunteers from 40 to 80 years of age. These patients underwent a standardized medical record review, including previous diagnosis and medications in addition to anthropometric, analytic, and radiologic studies, to assess personalized CV risk. Patients were recruited from 2019 to 2024 in a single university hospital in Spain. All patients underwent fasting lab tests including blood cell count, liver and kidney profile, CRP, lipid profile, glucose and glycohemoglobin (HbA1c) levels, triglycerides and glucose (TyG) and FIB-4 indexes, as well as full-body CT scan angiography, including CCTA. Patients unwilling to undertake any of the previously described procedures were excluded, as well as those with missing data from the prior assessments. Exclusion criteria comprised allergies to iodine contrast, a history of alcohol abuse, previously diagnosed viral or autoimmune liver disease, or advanced kidney disease. Patients with current or recent COVID-19 infection were excluded due to its potential to cause liver damage and capacity to alter liver function tests. Consequently, patients with liver impairment of tumor origin were also excluded for the same reason. Additionally, patients with CVD were excluded in this particular analysis to avoid confusion factors. This study was approved by the Institution Ethical Board (Ethical Board Reference 17/140).

### 2.2. Variables

Age, sex, smoking status, lipid profile, and blood pressure measurement according to the standards were used to calculate the 10 year-risk of a major cardiovascular event using SCORE2 [3] and SCORE2-OP [4]. Then, patients were distributed in low-to-moderate-risk (LMR), high-risk (HR), and very-high-risk (VHR) subgroups according to ESC guidelines [5].

Low-density lipoprotein cholesterol (LDL-C) excess was defined in patients with LDL-C levels exceeding the recommendations of the ESC guidelines: >100 mg/dL in LMR, >70 mg/dL in HR, and >55 mg/dL in VHR [5]. Low HDL-C was classified as levels < 40 mg/dL in men and <50 mg/dL in women. Hypertriglyceridemia was defined as triglycerides in plasma > 150 mg/dL. The TyG index was also calculated as previously described [27], with a cut-off > 8.8 points considered indicative of a high probability of metabolic syndrome development and, consequently, an increased risk of insulin resistance [28]. Elevated lipoprotein (a) levels were defined as values > 50 mg/dL, following the recommendations from previous studies and current clinical practice guidelines [5,21,22]. Type 2 diabetes mellitus was diagnosed when the fasting plasma glucose concentration was >125 mg/dL, HbA1c ≥ 6.5%, or there was use of anti-diabetic medication. Furthermore, hyperglycemia was considered when the fasting plasma glucose concentration was ≥100 mg/dL and HbA1c ≥ 5.7%. Hypertension was considered when blood pressure levels exceeded >140/90 mm Hg. An obesity assessment was performed using the BMI, while liver fibrosis was assessed using the FIB-4 formula, which includes age, alanine aminotransferase (ALT) (U/L), aspartate aminotransferase (AST) (U/L), and platelet count in blood (in 10^9^/L) [29]. Obesity was defined as a BMI > 30 kg/m^2^. Patients were considered to be at risk of liver fibrosis when the FIB-4 index was ≥1.3 points [18]. Metabolic syndrome was defined as the presence of at least 3 of the following 5 criteria: waist circumference > 94 cm in men and >88 cm in women; triglycerides ≥ 150 mg/dL; HDL-C < 40 mg/dL in men or <50 mg/dL in women; blood pressure ≥ 130/85 mm Hg; and fasting glucose ≥ 100 mg/dL or receiving treatment for it [30]. CRP levels above 0.2 mg/dL were considered as indicative of subclinical inflammation.

### 2.3. CCTA Image Acquisition and Analysis

CCTA was performed using a validated slice CT scanner Siemens Healthineers (Erlangen, Germany) with 140 kV tube voltage. A standard procedure of prospective electrocardiography-triggered scan was applied. One hour before the procedure, 50 mg of metoprolol was administered orally. Immediately before the scan, 0.4 mg of sublingual nitroglycerin was given to the patients, while continuously monitoring arterial pressure and heart rate. All CCTA images were reconstructed with a slice thickness of 3 mm. They were interpreted using the CAD-RADS scale [25] by an experienced radiologist who was blinded to the clinical information. The degree of stenosis was determined based on the maximum arterial narrowing observed throughout the studied area. “CV risk excess” is the main dependent variable analyzed. Risk excess was considered when patients at LMR had a CAD-RADS of 2 or higher, when patients at HR had a CAD-RADS of 3 or higher, and when patients at VHR had a CAD-RADS of 4 or higher according to previously published data on predicted CV events by CAD-RADS evaluation [26].

### 2.4. Statistical Analysis

Categorical variables were presented as percentages. Student’s *t* test, one-way ANOVA, or the X^2^-test were implemented to compare the baseline characteristics of study participants. A normality test was conducted using the Kolmogorov–Smirnov test, and for parameters that did not conform to normality (lipoprotein (a), AST, ALT, glucose, HbA1c, HOMA, FIB-4, and CRP), the Kruskal–Wallis test was used as a non-parametric alternative to ANOVA. Univariate logistic regression analyses and two-way ANOVA analyses were developed to analyze the ability of different CV risk factors to predict “CV risk excess”. Further multivariate regression models and two-way ANOVA analyses were developed to evaluate the effect and interactions between variables with a statistically significant capacity to detect CV risk excess. All statistical analyses were performed with SPSS version 20 (IBM Corp. Released 2011. IBM SPSS Statistics for Windows, Version 20.0. Armonk, NY, USA: IBM Corp.). All *p*-values are two-tailed and statistical significance was set at the conventional cut-off of *p* < 0.05.

## 3. Results

**Figure 1:**
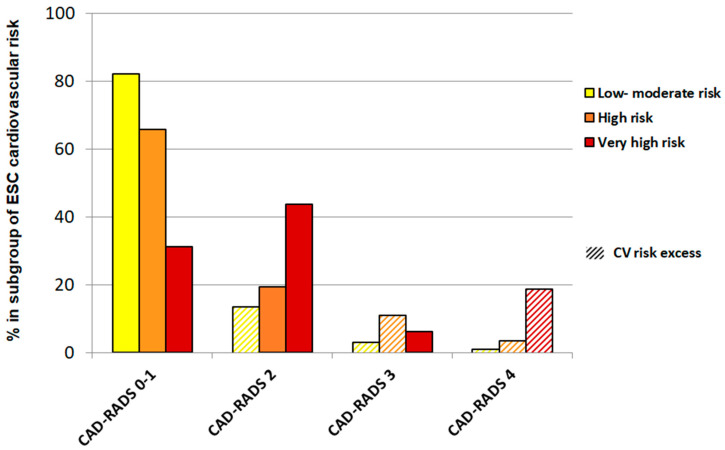
Distribution of cardiovascular risk among ESC risk subgroups according to CAD-RADS classification.

A total of 219 patients were included in this study. The baseline characteristics of the cohort are summarized in Table 1. According to ESC guidelines, patients were classified as either LMR (43.4%), HR (49.3%), and VHR (7.3%). The mean age was 58 years old and 14% were female. A total of 32% of the population were diagnosed with obesity at baseline, 45% had hypertension, and 11% had diabetes mellitus, with a higher prevalence observed in the groups with elevated CV risk (*p* < 0.01). In addition, patients at higher CV risk were more likely to have higher values of systolic and diastolic blood pressure, triglycerides, or fasting glucose. The mean FIB-4 index was 1.34 and 1.49 points in the HR and VHR groups, respectively, both exceeding the established cut-off point. No significant differences in lipoprotein (a) levels were found between the three groups (*p* = 0.988). The 18% of patients in the LMR group, 15% of patients in the HR group, and 19% of patients in the VHR group exhibited CV risk excess [Table 1 and Figure 1].

**Figure 2:**
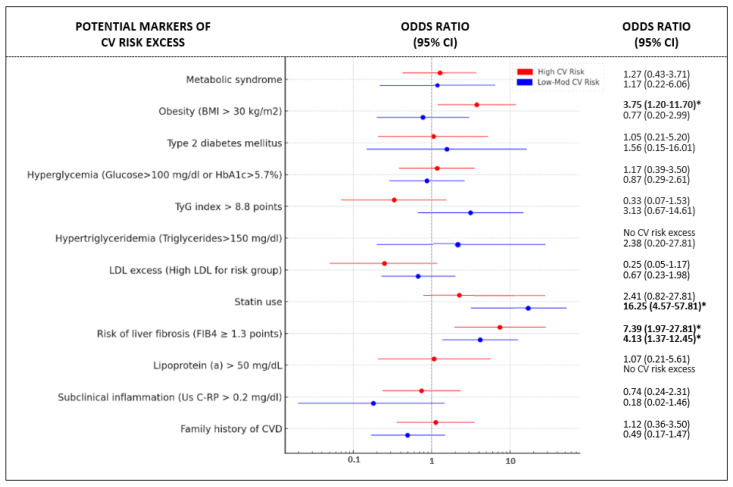
Forest plot of odds ratios (95% confidence intervals) for potential markers of cardiovascular risk excess, divided into ESC cardiovascular risk subgroups. The ratios are represented in a logarithmic scale. Abbreviations: CI—confidence intervals, mod—moderate, CV—cardiovascular, BMI—body mass index, HbA1c—glycohemoglobin, TyG—triglycerides and glucose, LDL—low-density lipoprotein, FIB4—fibrosis-4, Us C-RP—ultrasensitive C-reactive protein, CVD—cardiovascular disease. * Indicates statistical significance.

In the prediction of CVR excess, different CV risk parameters were evaluated with univariate logistic regression analysis. Statin use and liver fibrosis risk were associated with CV risk excess in the LMR group. Meanwhile, obesity and liver fibrosis risk showed statistical significance in the HR group [Figure 2]. These results persisted after age and sex adjustment [Supplementary Tables S1 and S2].

Interestingly, the combination of statin use and liver fibrosis risk (FIB-4 ≥ 1.3 points), and age- and sex-adjusted factors, showed a synergistic effect on CV risk excess prediction in the LMR group (*p* for interactions < 0.05) [Table 2]. Thus, patients were categorized into four subgroups: (i) “no statins use and FIB-4 < 1.3 points”, (ii) “no statins use and FIB-4 ≥ 1.3 points”, (iii) “statins use and FIB-4 < 1.3 points”, and (iv) “statins use and FIB-4 ≥ 1.3 points”. The presence of CV risk excess was observed in 4/49 patients in group (i), 0/20 in group (ii), 2/11 in group (iii), and 11/15 in group (iv). Only 2 of the 15 patients (13%) in group (iv) presented LDL-C values < 70 mg/dL. Patients with statin use and suspected liver fibrosis showed statistically significant differences in CV risk excess in comparison to the rest of the groups (F = 45.72 ± 4.24, *p* < 0.001). The reclassification analysis between the statin use group (model 1) and statin use + FIB-4 ≥ 1.3 points group (model 2) showed an IDI (integrated discrimination improvement) of 0.29, indicating a 29% improvement in model discrimination when incorporating FIB-4 ≥ 1.3 points as a risk factor. Additionally, the net reclassification index (NRI) was 0.57, suggesting that the new model improved the net reclassification by 57%. The number needed to intervene (NNI) in the group of statin use with liver fibrosis risk, to perform a CCTA for identifying CV risk excess, was 2 [Table 2].

Additionally, a modification of effect was found between obesity and liver fibrosis risk (*p* for interactions adjusted by age and sex < 0.05) [Table 2]. The combination between both variables provided a synergistic effect on CV risk excess prediction. Patients were stratified into four subgroups: (i) “no obesity and FIB-4 < 1.3 points”, (ii) “no obesity and FIB-4 ≥ 1.3 points”, (iii) “obesity and FIB-4 < 1.3 points”, and (iv) “obesity and FIB-4 ≥ 1.3 points”. The presence of CV risk excess was observed in 2/38 of patients in group (i), 3/25 in group (ii), 1/23 in group (iii), and 10/22 in group (iv). Patients with obesity and suspected liver fibrosis showed statistically significant differences in CV risk excess in comparison to the rest of the groups (F = 18.70 ± 1.95; *p* < 0.001). The reclassification analysis between the obesity group (model 1) and obesity + FIB-4 ≥ 1.3 points group (model 2) showed an IDI of 0.14, indicating a 14% improvement in model discrimination when incorporating FIB-4 ≥ 1.3 points as a risk factor in patients with obesity. Additionally, the NRI was 0.49, suggesting that the new model improved the net reclassification by 49%. The NNI in the group of obesity with liver fibrosis risk, to perform a CCTA for identifying CV risk excess, was 3 [Table 2].

**Figure 3:**
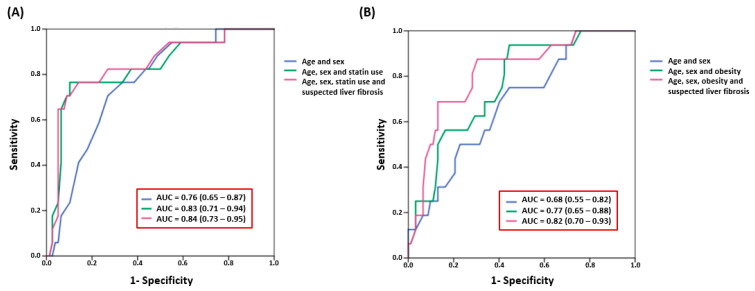
(**A**) Receiver operating characteristic curve of cardiovascular risk excess factors for the low-to-moderate-cardiovascular-risk category according to ESC guidelines. (**B**) Receiver operating characteristic curve of cardiovascular risk excess factors for the high-cardiovascular-risk category according to ESC guidelines.

Finally, a discrimination analysis was performed to evaluate the potential clinical role of these findings. In this context, a model of CV risk excess assessment for patients at LMR including statin use and risk of liver fibrosis measured by the AUROC provided an AUROC of 0.84 (95% CI 0.73–0.95) [Figure 3A]. Including obesity and risk of liver fibrosis in a model of CV risk excess for HR patients provided an AUROC of 0.82 (95% CI 0.70–0.93), as shown in Figure 3B. Both models showed a good discrimination performance.

## 4. Discussion

Accurate detection of subclinical disease is a cornerstone of preventive precision medicine [31]. The results of the present study demonstrate that patients with a greater-than-expected burden of atherosclerotic coronary disease can be identified by combining factors that are not considered in traditional risk stratification. Among these, liver fibrosis provides a synergistic capacity in detecting patients with excess risk when combined with pre-existing conditions such as statin use and obesity. Thus, these findings could shed light on patient CV risk stratification, incorporating imaging tests into the diagnostic work-up of CV risk in specific subgroups of patients.

Over the past few decades, and considering the findings from the Framingham studies [32], international organizations have supported various scales designed to categorize patients into risk groups based on demographic, anthropometric, clinical, and simple analytical data [3,4]. The goal has been to achieve a more-universal risk stratification and a certain order in recommending treatments for risk factor management, specifically those aimed at reducing LDL-C [5,7]. However, these detection and stratification methods express outcomes in the long term, such as mortality at 10 years and lifetime risk, which, although robust, lack adaptability to change and have limited significance in patient communication [33,34,35,36]. Additionally, the variables included in these scales are altered by treatments initiated based on scale results, complicating clarity in follow-up.

In this regard, the application of stratification methods linked to subclinical disease, specifically to subclinical atherosclerotic coronary disease burden, has demonstrated an additional capacity compared to traditional scales in detecting patients at risk for CVD [23,37,38]. Moreover, these methods provide an assessment framework with sufficient independence from the treatments initiated, allowing for personalized monitoring of treatment effects and adjustments. In this context, the evaluation of coronary stenosis through CCTA offers a highly accurate assessment of the coronary tree in patients, with the ability to detect those at excess risk [25]. The CAD-RADS classification has demonstrated the ability to provide independent prognostic information from other scales regarding all-cause mortality in patients with suspected CAD, where higher CAD-RADS classifications correlate with increased mortality risk [24,26,39]. Limitations, primarily related to radiation exposure, cost, and test availability, could be mitigated by selecting patients for whom this test would be particularly beneficial, as described in the subgroups identified by this study.

The findings from this study regarding the use of statins as a potential marker of excess risk may be controversial and warrant further explanation. Population studies, such as the SANTORINI study, have emphasized a concept that could be seen as contrary: the underutilization of lipid-lowering drugs in at-risk populations is one of the main reasons for the expansion of CVD burden [40]. Additionally, certain forums appear to be convinced of the benefits of population-wide statin treatment [41,42,43], even humorously fantasizing about the possibility of supplying them in drinking water. The results of this study do not imply that the use of statins increases CV risk. However, they underscore the importance of using statins appropriately, based on accurate stratification, to avoid masking high-risk patients within lower-risk subgroups. Inappropriate use of statins could deprive these individuals of proper stratification, tailored follow-up, and adjustments to their medication in accordance with their level of arterial disease. In contrast, treatment based on the assessment of subclinical atherosclerotic disease burden allows more objective and individualized care and could serve as a method to identify patients with underdosed use of statins.

Obesity is a well-established source of CV risk factors, including insulin resistance and atherogenic dyslipidemia, which are proven promoters of atherosclerosis [44,45,46]. However, weight excess alone is not included in traditional risk stratification methods in which obesity-derived mechanisms of atherogenesis, such as hypertension, dyslipidemia, and dysglycemia, are already accounted for [5]. Additionally, individuals with a high BMI but no metabolic disease, the so-called “healthy obese patients”, further reduce the discriminative power of obesity in this context [47]. Interestingly, the present research shows that patients with obesity and suspected liver fibrosis within the framework of SLD are more likely to have higher-than-expected CAD. A prior epidemiological study established a correlation between obesity, SLD, and CAD [15] and two prospective studies showed a linear relationship between the risk of SLD and the BMI [48]. Furthermore, significant weight loss has been shown to reduce the risk of both CAD and SLD [49]. All these points emphasize the shared pathophysiological pathways between these conditions [50]. Fibrosis biomarkers might reflect prolonged or more-severe exposure to metabolic alterations, indicating a higher degree of metabolic disease. In this context, previous studies suggest that fibrosis markers combined with other risk factors can significantly improve risk re-stratification [51]. In this scenario, the combination of obesity, measured by the BMI, and validated non-invasive fibrosis markers like FIB-4 provide a valuable approach for identifying a subgroup of patients with higher-than-expected coronary atherosclerosis. This combination highlights the benefit of coronary artery assessment in these individuals, enhancing preventive precision medicine.

This study has several limitations that should be considered. First, the cross-sectional design limits the ability to establish causal relationships between the observed variables and CV risk. Additionally, our definition of “CV risk excess” was based on discrepancies between predicted risk (SCORE2 or SCORE2-OP) and the actual burden of coronary atherosclerosis (CAD-RADS scores) rather than CV events. Consequently, long-term validation of this “CV risk excess” concept over a follow-up period of 10 years is needed to determine if it correlates with a higher incidence of CV events. However, the applicability of the results in clinical practice and the assessment of subclinical atherosclerosis burden, which has shown independent and longitudinal predictive power, partly mitigate these limitations [23]. Additionally, the study population consisted of volunteer patients interested in undergoing an exhaustive cardiovascular examination. This population assortment may introduce selection bias, as individuals with higher CV risk factors could have been more inclined to participate. Furthermore, we did not collect data on patients’ dietary habits or physical activity levels, which could have also contributed to their excess CV risk. Additionally, only 14% of the sample were women, which may not accurately represent the broader population and could influence outcomes related to sex differences in CV risk. These limitations were partially addressed by the methodology of the analyses, which included stratification by CV risk groups and adjustments in multivariable models. The sample size further reduces the statistical power to detect subtle associations or generalize the findings. Specifically, the small sample of the VHR group limited the analysis of CV risk factors in this subset of patients. Moreover, the use of surrogate measurements of obesity (instead of considering fat composition or distribution) [52,53] and liver fibrosis risk (without histological confirmation) are also potential limitations. However, the FIB-4 index is currently recommended in European MASLD guidelines as a useful screening tool for liver fibrosis in patients at metabolic risk [17,18], as the BMI is for obesity in WHO assessment recommendations [54], even though neither fully captures the complexity of liver fibrosis and obesity. We did not conduct a sensitivity analysis, as we prioritized the use of already-validated values. Further research is needed to establish new cut-off points for a more-personalized assessment.

The findings of this study emphasize the potential for improving CV risk stratification in routine clinical practice using clinically accessible resources. The proposed approach optimizes risk stratification, ensuring that patients with a greater likelihood of subclinical atherosclerosis are prioritized for further evaluation. Thus, these results highlight the need for tailored diagnostic work-up in the assessment of CV risk. Implementing these strategies could refine current CV risk assessment models and improve patient outcomes by directing personalized health policies to those who will benefit the most from intensified monitoring and intervention.

## 5. Conclusions

The combination of suspected liver fibrosis using the FIB-4 index with statin therapy and obesity in LMR and HR patients, respectively, is associated with further CAD than expected. Thus, the concurrence of these conditions might be considered a potential marker of “CV risk excess” and provide potential indications for image CAD assessment in asymptomatic patients.

## Figures and Tables

**Table 1:** General characteristics of patients classified by ESC guidelines into groups of cardiovascular risk.

Characteristics	Low-to-Moderate CV Risk n = 95	High CV Risk n = 108	Very High CV Risk n = 16	Total n = 219	*p*-Value
**Anthropometry**					
Age (years)	54.4 ± 1.3	59.1 ± 1.8	66.4 ± 3.0	57.9 ± 1.15	<0.001
Age group					0.006
<50 years	19 (20%)	22 (20.4%)	0 (0%)	41 (18.7%)	
50–70 years	72 (75.8%)	72 (66.7%)	11 (68.8%)	155 (70.8%)	
>70 years	4 (4.2%)	14 (13%)	5 (31.3%)	23 (10.5%)	
Sex (female)	23 (24.2%)	6 (5.6%)	2 (12.5%)	31 (14.2%)	0.001
**Obesity**	**20 (21.1%)**	**45 (41.7%)**	**5 (31.3%)**	**70 (32.0%)**	**0.007**
BMI mean (kg/m^2^)	27.0 ± 0.8	29.5 ± 0.8	29.6 ± 2.4	28.44 ± 0.6	<0.001
**Hypertension**	**27 (28.4%)**	**59 (54.6%)**	**12 (75.0%)**	**98 (44.7%)**	**<0.001**
SBP mean (mm Hg)	127.5 ± 3.0	141.8 ± 3.2	161.7 ± 9.1	137.0 ± 2.5	<0.001
DBP mean (mm Hg)	85.0 ± 2.0	88.7 ± 2.6	94.9 ± 5.8	87.5 ± 1.6	0.004
Antihypertensive drugs	25 (26.3%)	56 (51.9%)	11 (68.8%)	95 (43.4%)	0.001
**Dyslipidemia**	**40 (42.1%)**	**69 (63.9%)**	**8 (50.0%)**	**117 (53.4%)**	**0.008**
Total cholesterol (mg/dL)	194.7 ± 7.7	198.4 ± 8.5	184.3 ± 17.3	195.8 ± 5.5	0.419
HDL-C mean (mg/dL)	63.9 ± 2.9	53.4 ± 2.5	43.8 ± 3.0	57.3 ± 1.9	<0.001
Low HDL-C	2 (2.1%)	15 (13.9%)	4 (25.0%)	21 (9.6%)	0.002
Triglycerides mean (mg/dL)	80.3 ± 6.7	118.5 ± 17.8	132.5 ± 18.9	103.0 ± 9.7	<0.001
High triglycerides	15 (15.8%)	15 (13.9%)	2 (12.5%)	32 (14.6%)	0.901
LDL-C mean (mg/dL)	114.9 ± 7.1	125.1 ± 8.0	115.3 ± 15.7	120.0 ± 5.2	0.151
High LDL-C for risk group	63 (66.3%)	100 (92.6%)	16 (100%)	179 (81.7%)	0.391
Statin use	26 (27.4%)	41 (38.0%)	4 (25.0%)	71 (32.4%)	0.221
High LDL-C despite statin use	11/26 (42.3%)	34/41 (82.9%)	4/4 (100%)	49/71 (69.0%)	0.001
Lipoprotein (a) (mg/dL)	24.3 ± 5.9 ^a^	24.1 ± 5.9 ^b^	21.3 ± 13.5 ^c^	24.0 ± 4.0 ^d^	0.988
**Type 2 Diabetes Mellitus**	**4 (4.2%)**	**14 (13.0%)**	**6 (37.5%)**	**24 (11.0%)**	**<0.001**
Glycemia (mg/dL)	98.4 ± 4.8	103.6 ± 3.4	115.8 ± 9.5	102.3 ± 2.8	<0.001
Antidiabetic treatment	3 (3.2%)	11 (10.2%)	5 (31.3%)	19 (8.7%)	0.001
HbA1c (%)	5.48 ± 0.12	5.66 ± 0.11	6.14 ± 0.46	5.6 ± 0.08	0.001
HOMA-IR	3.4 ± 2.8	3.0 ± 0.4	4.8 ± 2.6	3.3 ± 1.3	<0.001
Insulin µU/mL	8.4 ± 1.1	11.7 ± 1.5	15.6 ± 5.9	10.5 ± 1.0	<0.001
**TyG Index**	8.19 ± 0.09	8.55 ± 0.11	8.89 ± 0.20	8.42 ± 0.07	<0.001
TyG > 8.8 points	8 (8.4%)	30 (27.8%)	9 (56.3%)	47 (21.5%)	<0.001
**Smoking status**					**<0.001**
Never smokers	51 (53.7%)	44 (40.7%)	3 (18.8%)	98 (44.7%)	
Previous smokers	37 (38.9%)	31 (28.7%)	3 (18.8%)	71 (32.4%)	
Active smokers	7 (7.4%)	33 (30.6%)	10 (62.5%)	50 (22.8%)	
**Metabolic syndrome**	**10 (10.5%)**	**42 (38.9%)**	**8 (50.0%)**	**60 (27.4%)**	**<0.001**
**C-reactive protein (mg/dL)**	**0.21 ± 0.07**	**0.23 ± 0.04**	**0.29 ± 0.15**	**0.22 ± 0.04**	**0.081**
**Suspected liver fibrosis (FIB-4 ≥ 1.3)**	**35 (36.8%)**	**47 (43.5%)**	**8 (50.0%)**	**90 (41.1%)**	**0.473**
FIB-4 mean	1.20 ± 0.08	1.34 ± 0.11	1.49 ± 0.29	1.29 ± 0.07	0.137
AST (U/L)	24.49 ± 16.03	25.09 ± 17.63	21.38 ± 8.99	24.56 ± 16.50	0.214
ALT (U/L)	25.42 ± 24.46	27.54 ± 29.22	23.63 ± 18.81	26.33 ± 26.60	0.635
Platelets (10^9^/L)	234.94 ± 100.03	235.24 ± 103.59	221.81 ± 128.85	234.13 ± 103.80	0.628
**Coronary CT angiography**					**<0.001**
CAD-RADS 0	47 (49.5%)	28 (25.9%)	2 (12.5%)	77 (35.2%)	
CAD-RADS 1	31 (32.6%)	43 (39.8%)	3 (18.8%)	77 (35.2%)	
CAD-RADS 2	13 (13.7%)	21 (19.5%)	7 (43.8%)	41 (18.7%)	
CAD-RADS 3	3 (3.2%)	12 (11.1%)	1 (6.3%)	16 (7.3%)	
CAD-RADS 4	1 (1.0%)	4 (3.7%)	3 (18.8%)	8 (3.7%)	
**CV risk excess**	**17 (17.9%)**	**16 (14.8%)**	**3 (18.8%)**	**36 (16.4%)**	**0.812**

Abbreviations: CV—cardiovascular, BMI—body mass index, SBP—systolic blood pressure, DBP—diastolic blood pressure, HDL-C—high-density lipoprotein cholesterol, LDL-C—low-density lipoprotein cholesterol, HbA1c—glycohemoglobin, HOMA-IR—homeostasis model assessment of insulin resistance, TyG—triglycerides and glucose, FIB-4—fibrosis-4, AST—aspartate aminotransferase, ALT—alanine aminotransferase, CAD-RADS—coronary artery disease reporting and data system. ^a^ n = 83, ^b^ n = 92, ^c^ n = 12, ^d^ n = 187. Bold is used to clearly differentiate the data categories in the table, making the structure more organized.

**Table 2:** Evaluation of the association between statin use and suspected liver fibrosis in patients at low-to-moderate cardiovascular risk and between obesity and suspected liver fibrosis in patients with high cardiovascular risk according to ESC guidelines for predicting excess cardiovascular risk.

Low–Moderate CV Risk (n = 95)	Risk Excess, n (%) [n = 17]	*p*	*p* Adjusted by Age and Sex	High CV Risk (n = 108)	Risk Excess, n (Total %) [n = 16]	*p*	*p* Adjusted by Age and Sex
**Model 1**				**Model 1**			
No statin use (n = 69)	4 (6%)	<0.001	<0.001	No Obesity (n = 63)	5 (8%)	0.017	0.017
Statin use (n = 26)	13 (50%)			Obesity (n = 45)	11 (24%)		
FIB-4 < 1.3 (n = 60)	6 (10%)	<0.01	0.048	FIB-4 < 1.3 (n = 61)	3 (5%)	0.008	0.018
FIB-4 ≥ 1.3 (n = 35)	11 (31%)			FIB-4 ≥ 1.3 (n = 47)	13 (25%)		
**Interaction** Statin use × FIB-4		<0.001	<0.001	**Interaction** Obesity × FIB-4		0.008	0.011
**Model 2**				**Model 2**			
No statins and FIB-4 < 1.3 (n = 49)	4 (8%)	<0.001	<0.001	No obesity and FIB-4 < 1.3 (n = 38)	2 (5%)	0.011	0.001
Statins and FIB-4 < 1.3 (n = 11)	2 (18%)			Obesity and FIB-4 < 1.3 (n = 23)	1 (4%)		
No statins and FIB-4 ≥ 1.3 (n = 20)	0 (0%)			No obesity and FIB-4 ≥ 1.3 (n = 25)	3 (12%)		
Statins and FIB-4 ≥ 1.3 (n = 15)	11 (73%)			Obesity and FIB-4 ≥ 1.3 (n = 22)	10 (45%)		

Abbreviations: CV—cardiovascular.

## Data Availability

The original contributions presented in this study are included in the article/supplementary material. Further inquiries can be directed to the corresponding author.

## Supplementary Materials

The following supporting information can be downloaded at https://www.mdpi.com/article/10.3390/jcm14041218/s1. Table S1: Combined analysis of statin use and suspected liver fibrosis in patients at low-to-moderate cardiovascular risk according to ESC guidelines for predicting excess cardiovascular risk; Table S2: Combined analysis of obesity and suspected liver fibrosis in patients at high cardiovascular risk according to ESC guidelines for predicting excess cardiovascular risk.
