# Supplementary material for: Clinically Accessible Liver Fibrosis Association with CT Scan Coronary Artery Disease Beyond Other Validated Risk Predictors: The ICAP Experience
Source: J Clin Med. 2025 Feb 13;14(4):1218. doi: 10.3390/jcm14041218 (PMC11856594; doi:10.3390/jcm14041218)
Supplement: Supplementary file 1 [file jcm-14-01218-s001.zip › jcm-3401302-supplementary.pdf]

Supplementary table 1. Combined analysis of statin use and suspected liver fibrosis in patients at low to moderate cardiovascular risk according to ESC guidelines for predicting excess cardiovascular risk.

Variable	CV Risk excess OR (95 % CI)	p- value
Model 1 – Adjusted by age and sex		
Statin use	11.39 (2.93-44.28)	<0.001
Model 2 – Adjusted by age and sex		
Statin use		
Statin use x Suspected liver fibrosis	9.81 (1.32-72.72)	0.026
Model 3 – Adjusted by age and sex		
Statin use & Suspected liver fibrosis	23.59 (4.82-115.46)	<0.001

Supplementary table 2. Combined analysis of obesity and suspected liver fibrosis in patients at high cardiovascular risk according to ESC guidelines for predicting excess cardiovascular risk.

Variable	Risk excess OR (95 % CI)	p
Model 1 – Adjusted by age and sex		
Obesity (BMI > 30 kg/m^2)	3.65 (1.09-12.21)	0.036
Suspected liver fibrosis (FIB-4 ≥ 1.3)	4.81 (1.06-21.94)	0.042
Model 2 – Adjusted by age and sex		
Obesity	0.67 (0.07-6.33)	0.723
Obesity x Suspected liver fibrosis	11.81 (1.20-116.03)	0.034
Model 3 – Adjusted by age and sex		
Obesity -/Suspected fibrosis -	Ref.	-
Obesity +/Suspected fibrosis -	0.93 (0.08-11.07)	0.951
Obesity -/Suspected fibrosis +	1.74 (0.23-12.92)	0.589
Obesity +/Suspected fibrosis +	10.89 (1.87-63.45)	0.008
Model 4 – Adjusted by age and sex		
Obesity & Suspected liver fibrosis	8.42 (2.44-29.02)	0.001
